# Scaling of contact networks for epidemic spreading in urban transit systems

**DOI:** 10.1038/s41598-021-83878-7

**Published:** 2021-02-23

**Authors:** Xinwu Qian, Lijun Sun, Satish V. Ukkusuri

**Affiliations:** 1 Department of Civil, Construction and Environmental Engineering, The University of Alabama, Tuscaloosa, AL 35487 USA; 2 Department of Civil Engineering, McGill University, Montreal, QC H3A 0G4 Canada; 3 Lyles School of Civil Engineering, Purdue University, West Lafayette, IN 47907 USA

**Keywords:** Civil engineering, Complex networks

## Abstract

Improved mobility not only contributes to more intensive human activities but also facilitates the spread of communicable disease, thus constituting a major threat to billions of urban commuters. In this study, we present a multi-city investigation of communicable diseases percolating among metro travelers. We use smart card data from three megacities in China to construct individual-level contact networks, based on which the spread of disease is modeled and studied. We observe that, though differing in urban forms, network layouts, and mobility patterns, the metro systems of the three cities share similar contact network structures. This motivates us to develop a universal generation model that captures the distributions of the number of contacts as well as the contact duration among individual travelers. This model explains how the structural properties of the metro contact network are associated with the risk level of communicable diseases. Our results highlight the vulnerability of urban mass transit systems during disease outbreaks and suggest important planning and operation strategies for mitigating the risk of communicable diseases.

## Introduction

The rapid growth of population and activity intensity in megacities have propelled an evolutional shift of urban mobility from individual-centric travel to sustainable urban mobility. This substantial shift concerns environment, energy consumption, equity, among others^1–4^. Lying at heart for promoting sustainable travel is our understanding of the interplay among urban form, transportation system, and human mobility. Research across a diverse stream of studies and data sources have shown the scaling properties of individual mobility^5–8^: the vast majority of people travel between a few popular locations and their travel distances are bounded by the scale of the city and its transportation systems.

One indispensable component of urban transportation is the mass transit system, which is so far the only sustainable solution to serve urban mobility needs on a large scale. In 2018, public mass transit served over 53 billion passengers worldwide. The three busiest metro systems reached the daily ridership of 9.48 million (Tokyo), 6.49 million (Moscow) and 5.6 million (Shanghai)^9^, respectively. While these systems allow a large number of commuters to travel efficiently, they also result in high population density within close proximity for long durations. These features establish an environment conducive to the spread of communicable diseases^10–12^. In particular, pathogens of infectious travelers can migrate to adjacent travelers through droplets and airborne transmissions, resulting in secondary infections during travel. Public mass transit and the underlying travel patterns are becoming an essential catalyst for influenza pandemics and may greatly accelerate the spreading pace of communicable diseases and thus increase the intensity of disease outbreaks in megacities. Despite acknowledging the linkage between human mobility and the spread of infectious disease^8,13–18^, current models do not understand the nexus on how the structure of physical contacts/encounters among individuals—which in turn enable the transmission of communicable diseases—are affected by the interaction between human mobility and physical infrastructures.

An attractive approach to address the challenge is to construct the contact networks during travel and then embed the disease percolation process among individual travelers into the contact networks. Recent advances in complex network theories and epidemic modeling have established a striking connection between network structure and disease dynamics^19–24^ and the epidemic spreading was modeled on various mobility scales including airline network^17,25,26^, ground transportation network^14,16^ and river network^15,27^. While mobility networks represent mediate channels for epidemic outbreaks, other studies focused on correlating disease dynamics with direct human interactions as contact networks at activity locations^12,22,28–30^. More recently, the prevalence of individual-level mobility data and disease record data with fine granularity also opens new opportunities for directly linking the observed dynamics of infectious diseases with the human mobility patterns of the corresponding periods. For instance, the human mobility data was overlayed with insurance claims data to investigate the drives of seasonal influenza^31^, track and predict the fate of the dengue^32^, real-time prediction of Zika outbreak^33^, and measure the effectiveness of control measures for the recent COVID-19 outbreak^34–36^. The availability of data also motivated initial attempts on exploring the risk of infectious diseases in public transportation systems, where the transit smart card data and passenger demand data are used to restore individual trip sequences, construct potential encounter networks among travelers and simulate the outbreaks of infectious diseases on the encounter networks^37–39^. These studies connected the disease percolation process with either mobility networks or contact networks, and the data and the networks are mainly used to deliver descriptive and predictive analyses. However, such analyses are reactive and may not guide the development of proactive measures against the threat of infectious diseases. The linkage on how contact networks are generated as a function of human mobility and the transportation infrastructures is still missing. Meanwhile, such an interplay has significant implications on systematically keeping infectious people from engaging in daily activities and consequently stopping the epidemic from the source.

**Figure 1:**
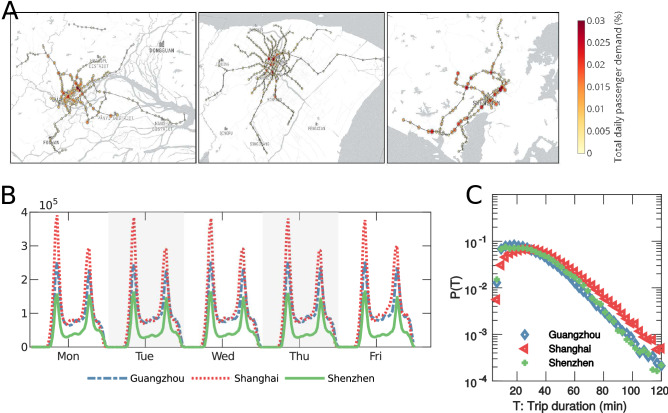
Summary characteristics of the travel pattern within the metro networks. (**A**) Spatial layout of the metro networks of the three cities (from left to right: Guangzhou, Shanghai, Shenzhen). The satellite images for the metro network layout were created using mplleaflet library in Python3, which can be accessed at https://github.com/jwass/mplleaflet. (**B**) Temporal distribution of metro ridership obtained from the smart card transaction data. (**C**) Probability density of function of metro trip duration T. We observe that the distributions metro trip duration of all three cities follow exponentially decaying tails ($p(T)\propto e^{-T/\lambda }$). For Guangzhou, we find that trips with $T>50\hbox {min}$ can be well fitted with $\lambda =13.78\hbox {min}$. For Shanghai, trips with $T>50\hbox {min}$ can be well fitted with $\lambda =16.59\hbox {min}$. For Shenzhen, trips with $T>50\hbox {min}$ can be well fitted with $\lambda =13.43\hbox {min}$.

To close the gaps, this study aims to characterize how human mobility shapes contact networks during travel and how it subsequently affects the threshold of disease percolation among individual travelers. In previous studies, contact networks were either of high-resolution for small systems (e.g., at conference^28^ and school^12,29^) or of low-resolution for large systems by simulating from survey data^22,30^. Here we construct high-resolution contact networks for city-wide transit systems by leveraging smart card data from three major cities in China: Guangzhou, Shanghai, and Shenzhen (see SI Appendix S1 for a detailed description of the data). We focus on the metro system, the most used public transit mode, to rebuild the contact networks among metro travelers, but the approach is broadly applicable to other transit systems. The three cities are of drastically different scales and distinct metro network layouts (Fig. 1A). Specifically, Shanghai has the largest population, metro system, and the highest metro passenger volume (over 4 million daily records), followed by Shenzhen (2.1 million) and Guangzhou (1.6 million). The metro ridership of the three cities presents highly regular and recurrent patterns during weekdays with prominent travel peaks (Fig. 1B), which implies a large number of daily commuters and repeated metro visits. The large-scale trip data, as the result of intensive daily activities in metro systems, allow us to directly probe the representative mobility patterns of metro travelers in these cities (see Fig. 1C). Despite distinct network layouts, size, and trip demand, the mobility patterns of the three cities are observed to be strikingly similar. We find that there is a large number of travelers with travel time under 50 minutes, and the number of travelers decays exponentially with increasing trip length. This finding holds true across all three cities, with Shanghai having a lower decay rate (16.59min) and the decay rates for Guangzhou (13.78min) and Shenzhen (13.43min) being almost identical. This indicates that trip lengths in metro systems are bounded by the size of the metro network, which reflects the scale of a city, and the results are also consistent with the reported metro mobility patterns in other cities^8,40^. The finding also provides strong evidence to support the universality of human mobility within public transit, where the travel time follows the exponential distribution with the decay rate being proportional to the scale of the city. As physical encounters are driven by human mobility, this motivates us to investigate the possible existence of scaling laws for the contact patterns in public mass transit networks, as the results of the universal mobility patterns.

**Figure 2:**
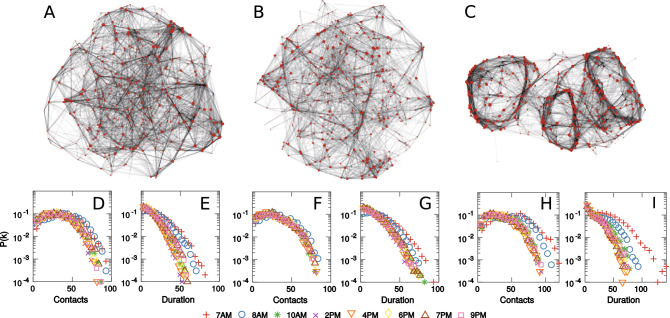
Simulated MCNs with 500 nodes and the unweighted and weighted degree distributions of simulated MCNs with 1000 nodes. (**A**)–(**C**) visualizes the layouts of the simulated MCNs in Guangzhou, Shanghai and Shenzhen. In the visualization, larger node size reflects higher node degree and the transparency of the link is proportional to the duration of contact. (**D**), (**F**) and (**H**) present the probability density function of the unweighted degree distributions of Guangzhou, Shanghai and Shenzhen. (**E**), (**G**) and (**I**) present the probability density function of weighted degree distributions of Guangzhou, Shanghai and Shenzhen.

## Results

### Metro contact network

Smart card data only provide entry and exit information on a trip. To gain insights into how travelers come in contact with others during travel, we develop a simulation model based on the observed metro network layout, demand profile, and mobility patterns. The simulation constructs high-resolution metro contact networks (MCNs) by first sampling passenger arrivals at each metro station and their trip destinations, then calculating if two individuals will come into contact based on their trip profiles, and finally assigning expected contact duration between each pair of individuals (The detailed description of the simulation is presented in Methods). The inputs to the simulation are the number of travelers (*N*), the time period of interest, the operational timetable and the metro network layout. The simulation then produces a $N\times N$ matrix describing the physical contact pattern between each pair of individual travelers. In particular, each positive entry of the matrix denotes the expected contact duration between two travelers within *effective transmission range*, e.g., two individuals are of close proximity so that the airborne transmission of a communicable disease is likely. For typical droplet transmission, the effective range is less than 3 feet while certain diseases such as SARS may reach 6 feet^41^.

**Table 1:** Summary statistics of the generated MCNs with 1000 nodes.

	$\langle k \rangle$	Clustering coefficient	Diameter	CPL
Guangzhou	35.09	0.49	7.2	2.79
Shanghai	29.14	0.46	7.6	2.89
Shenzhen	41.59	0.54	6	2.78

The MCNS correspond to the travel pattern during 8–8:30 AM on weekdays, and the statistics are measured using unweighted MCNs. Results are averaged from 10 random realizations.

We then visualize the structure of the MCNs by simulating a sample realization for each of the cities during 8–8:30 AM, and we set the number of travelers to 500 for better visibility of the network structure (Fig. 2A–C). We observe that the MCNs are visually different among the cities, which is due to the differences in metro network layouts. But these MCNs also share several structural commonalities, including local clusters of nodes and the discrepancies of node degree.

To gain a better understanding of their structural properties, for each city, we further generated MCNs from 500 to $10^4$ nodes and the changes in structural properties with increasing network size are summarized in SI Appendix S2.1. We observe that there are fewer hubs in the MCNs as opposed to the scale-free networks. Instead, there are a large number of nodes with low to medium degree. We further confirm the structural similarities of the MCNs among the three cities by different quantitative metrics, as shown in Table 1. All the MCNs are observed to have high node degree heterogeneity and high clustering coefficient, and have small network diameter and characteristic path length (CPL). These properties are statistically different from the metrics of their random counterparts (see SI Appendix S2.2), which corroborates the distinct structural properties associated with MCNs. These confirm that MCNs are a type of small-world network^42^, and this observation leads to significant implications in the context of disease percolation. Specifically, the outbreak of an exceedingly infectious disease may quickly synchronize among all the travelers because of the small-world property, and therefore the metro system becomes highly vulnerable. But unlike many real-world networks, we report that several network metrics (e.g., clustering coefficient, diameter and assortativity) are invariant to the size of the MCNs (Table S3–S5). Instead, they are determined by the metro network layout and human mobility patterns. In this regard, MCNs, like many other real-world contact networks in school, conference sites and major activity locations, should be regarded and studied as the product of the interactions between human mobility and physical infrastructure.

By plotting the degree distributions of the number of contacts and contact duration for metro travelers (Fig. 2D–I), we find an even more remarkable similarity among the MCNs. Despite the differences in metro network layouts, visualizations and statistics of the simulated MCNs, the degree distributions are found to follow a similar distribution across the three cities, and such observation is also valid for different time periods of the day. In particular, the unweighted degree distributions of the MCNs show a large number of nodes of low to medium degrees (e.g., degree smaller than 50) and the node degrees within this range are found to be nearly uniformly distributed. But with an increasing number of contacts and length of contact duration, the tails of the node degree distributions are found to decay exponentially, similar to the observations for metro mobility patterns. In addition, the rates of decay are found to be time-dependent and also differ among the cities. We also find that, in all three cities, both unweighted and weighted degree distributions during morning peak hours (7 AM and 8 AM) are slightly different from those in other time periods. This can be understood from the mobility patterns of the metro travelers during the time, where morning commuters are more likely to travel in the same direction (e.g., from home to work), thus increasing the number of contacts and the contact duration. Such an observation is later confirmed in our generation model, where morning commuters present a higher degree of similarity in terms of their trip patterns. These observations lead to the conjecture of a universal mechanism underlying the contact of metro travelers, and we explore the mechanism in more depth in the following sections.

### Disease dynamics in contact network

With reconstructed contact networks, the risk of communicable diseases can be quantified by modeling the dynamics of disease percolation among individual travelers. We introduce an individual-based model (IBM) following^43^. To characterize disease dynamics within the contact network, the classical susceptible-infectious-susceptible (SIS) process is embedded in the IBM over MCNs. Unlike previous studies^19,22^, this framework does not require nodes and transmission between nodes to be homogeneous, which allows us to model heterogeneous infectious rates due to the varying contact duration. Denote the probability that node *i* is infected at time *t* as $p_{i,t}$ and the recovery rate as *r*, we have

1 $$\begin{aligned} p_{i,t}=1+p_{i,t-1}(q_{i,t}-r)-q_{i,t},\forall \,i\in V \end{aligned}$$

where $q_{i,t}$ represents the probability that node *i* is in S at time *t*, which depends on that all its neighbors $j\in {\mathcal {N}}(i)$ are either in S or in I but fail the transmission:

2 $$\begin{aligned} q_{i,t}=\prod _{j\in {\mathcal {N}}(i)} (1-p_{j,t}+(1-\beta _{i,j})p_{j,t}) \end{aligned}$$

In the equation, $\beta _{i,j}=\beta t_{i,j}$ represents the transmission rate between node *i* and *j*, which takes the product of per unit time disease transmission strength $\beta$ and the contact duration $t_{i,j}$. With *N* such nodes, we arrive at a nonlinear dynamic system (see SI Appendix S3.2) with two equilibrium states: (1) the disease-free equilibrium (DFE) where all individuals are in S state (e.g., $p_{i,t}=0$) and (2) the endemic equilibrium where a positive proportion of individuals are in I state. The asymptotic stability of the DFE relies on the network-specific critical threshold $\delta$ that is associated with the largest eigenvalue of the adjacency matrix of MCNs. And we show that the critical threshold is upper bounded by the largest node degree in MCNs as:

3 $$\begin{aligned} \delta \le \max _i \sum _j \beta _{i,j}-r+1=\bar{\delta } \end{aligned}$$

As a consequence, if $\bar{\delta }< 1$, the disease is guaranteed to go extinct while the disease may be endemic with $\bar{\delta }>1$. Since $\beta$ and *r* are endogenous parameters, the risk level that pertains to a specific disease primarily depends on the value of $\max _i \sum _j\beta _{i,j}$. Such finding subsequently builds the essential connection between the vulnerability of public mass transit with the degree distribution of its contact networks and identifies the impact of the structural property of MCNs on disease threshold in transit systems. The observation provides two immediate implications. First, we can verify that the risk level of an MCN is driven by the riskiest individual who has the highest number of contacts or contact duration, which concerns the tail pattern of MCNs unweighted and weighted degree distributions. Second, by removing the riskiest individual, the next riskiest person may have a similar risk level according to the revealed degree distributions (Fig. 2D–I). This highlights the difficulties in improving MCN’s vulnerability and stopping the disease during outbreaks.

In addition to the IBM model, we also build an equivalent OD-based mean-field (ODMF) approach that models the disease dynamics on the passenger flow level between each pair of metro stations (SI Appendix S4). Note IBM is computationally expensive due to the construction of MCNs, and the ODMF can be used to approximately probe the system-wide disease dynamics for the real number of metro travelers.

### Disease control strategies

**Figure 3:**
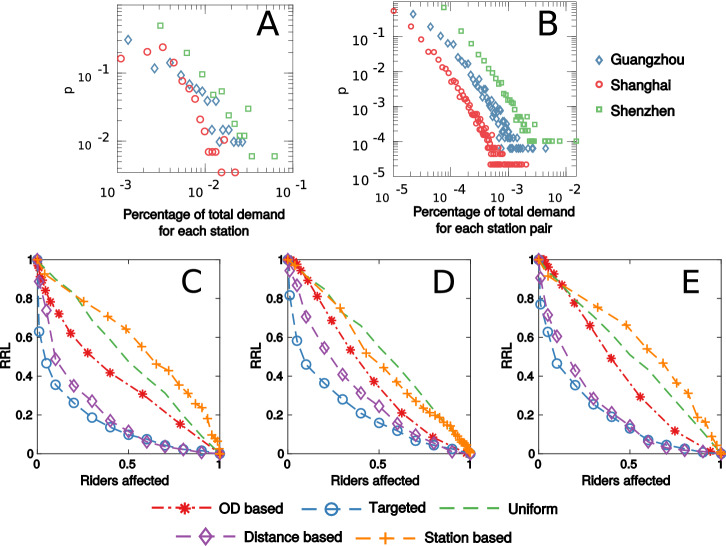
Distribution of travel demand and the effectiveness of different control strategies for the MCNs of the three cities. (**A**) presents the probability density function of the trip demand at the station level. (**B**) presents the probability density function of the trip demand of each pair of stations. (**C**), (**D**) and (**E**) visualize the effectiveness of OD based, targeted, uniform, distance based and station based control strategies for each city. The effectiveness of control strategies is compared with the proportion of trip demand affected by the corresponding strategy.

The best practice for controlling the disease is to immunize travelers through vaccination and quarantine^44^. We next explore the effectiveness of five immunization strategies with the percentage of individuals immunized as a control parameter. *Origin-Destination pairs (OD)* based and *station-based* approaches represent population control that immunizes a portion of travelers commuting between a pair of stations or originating from a station. These two control strategies are motivated by the observation that fewer than 20% of the stations produce over 80% of travel demand (Fig. 3A), and more than 80% of the metro commuters are associated with fewer than 20% of station pairs(Fig. 3B). These findings suggest that population control may yield satisfactory results in reducing the risk level by focusing on populated stations and trip pairs, as these travelers are likely to have more number of contacts. On the other hand, we also consider *uniform*, *targeted* and *distance based* approaches that are individual-centered methods. The uniform strategy immunizes randomly selected travelers, the targeted strategy iteratively removes travelers of the longest contact duration, and the distance-based strategy aims at immunizing travelers of the longest travel time. Specifically, the targeted method is reported to be most effective in the complex network literature^19,21^. The effectiveness of these control strategies is then examined based on the relative risk level (RRL), which measures the reduction in $\max _i \sum _j\beta _{i,j}$ with an increasing number of immunized travelers.

Our results suggest that the most effective method is the targeted immunization followed by the distance-based method and the OD based method. All three are superior to the uniform immunization. This finding is consistent across the three cities (Fig. 3C–E). For the targeted immunization, we observe a 27% reduction in RRL by immunizing the top 1% riskiest individuals, and a 60% reduction in RRL can be achieved by removing the top 10% riskiest travelers. On the other hand, the station based method is found to be less effective than the uniform policy in two of the three cities. The primary reason is that populated stations may not be origins of travelers with a high number of contacts and contact duration. Unfortunately, it is usually impracticable to identify the risk level of a traveler before the trip, which is the major barrier for the implementation of targeted immunization. As effective alternatives, we may introduce the distance-based and OD-based strategies by tracking the historical trips of an individual from her smart card. The most practical control strategies are the station based and uniform strategies, but neither is shown to be effective enough. These results highlight the challenges for stopping the spread of the disease in transit systems. It is therefore important to devise better strategies for the operation of metro systems andimporve the structure of the metro networks.

### A generation model for MCNs

Here we develop and validate a simple generation model for MCNs. The MCN to be generated is a scale-dependent network where the degree distribution is a function of the total number of travelers in the network. We observe that the travelers’ mobility patterns follow the exponential distribution while the contact degree distributions also decay exponentially. Following our discussion on the MCN structure, we hypothesize that (1) contacts are driven by travelers’ mobility patterns, and (2) the probability of two travelers getting into contact is bounded by their mobility in the metro network and also the layout of the metro network. We focus on investigating a universal generation model with individuals’ mobility pattern as the input and we consider that the number of contacts is proportional to the total travel time $t_i$ of each traveler. Recall that the degree distribution is found to vary across time and city. We thereafter introduce two variables: $\alpha$ captures the impact from metro network layout and $\gamma _t$ models the temporal characteristics of travelers’ mobility. We consider the expected total number of contacts experienced by *N* travelers as:

4 $$\begin{aligned} C= \sum _i^N \alpha t_i^{\gamma _t} (N-1) \end{aligned}$$

Equation 4 accounts for the scale-dependent nature by including *N* on the right-hand side and $\alpha t_i^{\gamma _t}$ determines the rate that a commuter of travel time $t_i$ will meet other $N-1$ travelers in the system. And $t_i^{\gamma _t}$ refers to the rescaled travel time which depends on the temporal trip similarity among travelers. This is motivated by the fact that different times of day will result in riders heading to various destinations and $\gamma _t$ therefore measures how similar their destinations are. Consider $M=2C$ as the total number of stubs (half-edges) in MCNs, we can derive the probability that a node is of degree *k* as (see SI Appendix S5 for derivation details and Fig. S8 on empirical evidence that supports *M*):

5 $$\begin{aligned} p(k)=\sum _{i=1}^N \frac{(Mw_i)^ke^{-Mw_i}}{k!}p(t_i) \end{aligned}$$

with $w_i = 2\alpha t_i^{\gamma _t} (N-1)/M$ being the probability that a randomly selected stub is attached to node *i*. Given the PDF in Eq. (5), the MCNs can then be generated following the configuration model by first sampling the degree sequence $K=\{k_1,k_2,...,k_N\}$ from *p*(*k*), then randomly selecting and connecting a pair of stubs until all stubs are exhausted. For each pair of matched stubs between node *i* and *j*, we further assign the weight $d_{ij}\propto \min (t_i^{\gamma _t},t_j^{\gamma _t})$ as the edge weight and obtain the weighted degree distribution.

To calibrate $\alpha$ and $\gamma _t$, we perform cross-validation to determine the optimal $\alpha$ for each city and the corresponding $\gamma _t$ at each time interval, with the objective to minimize the Kolmogorov–Smirnov (KS) statistics between the CDFs of the generated and simulated MCNs for both unweighted and weighted degree distributions (see SI Appendix S5.1). To validate the correctness of the generation model, we conduct two-sample KS tests to compare the CDFs of unweighted and weighted degree distributions between generated and simulated MCNs. The null hypothesis is that the two data samples for comparison are drawn from the same continuous distribution.

**Figure 4:**
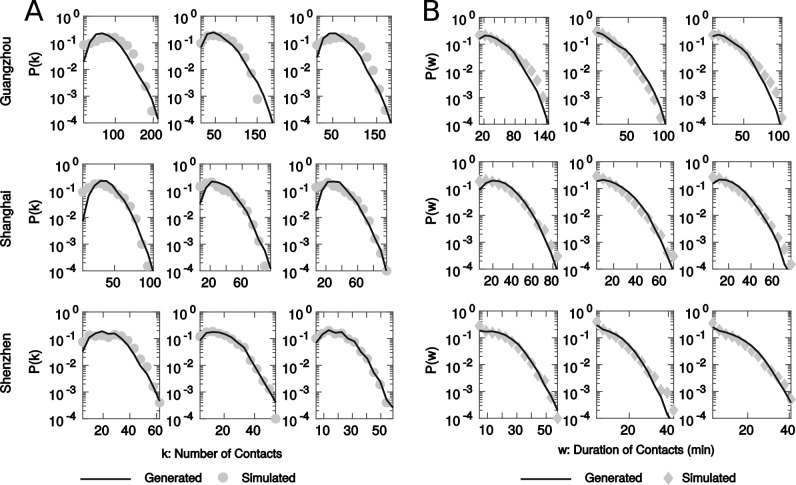
Visualization for the goodness of fit of the generated MCNs as compared to the simulated MCNs from the smart card data. Each row corresponds to the results of the same city and each column corresponds to the results from a particular time of the day. (**A**) Fitting results of the probability density function for the unweighted degree distributions. (**B**) Fitting results of the probability density function for the weighted degree distributions. All results are obtained from the average performance of 50 generated MCNs using the optimal $\gamma _t$ and from the average of 50 simulated MCNs. Each MCN has 1000 nodes. All scenarios fail to reject the null hypothesis of the KS test with very high p-values, where the summary statistics of the KS test and calibrated model parameters are shown in Table 2.

The validation results are summarized in Table 2, and we also visualize the fitting of the generated MCNs in Fig. 4. We observe that for all experiments, we fail to reject the null hypothesis for the two-sample KS test with the lowest p-value among these cases being 0.742. Even this lowest value is way above the significant threshold for rejecting the null hypothesis (0.05), and in most cases, the *p* value is greater than 0.95 for both weighted and unweighted distributions. The statistics along with the goodness of fit in Fig. 4 are indicative that the proposed generation function well captures the underlying mechanisms that govern the meetings of passengers and the duration of exposures during their travels in metro systems. More importantly, the validation of the generation model in three cities provides strong evidence for the existence of a universal rule that shapes the contacts among travelers in transit networks.

**Figure 5:**
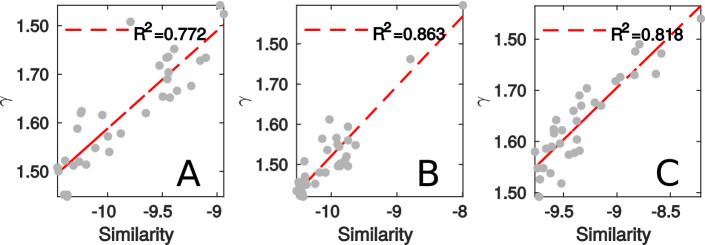
Comparison between the calibrated parameter $\gamma _t$ and trip similarity index for (**A**) Guangzhou, (**B**) Shanghai and (**C**) Shenzhen.

**Table 2:** Summary of fitted results and model parameters from KS test for three cities.

Time	8 AM	12 PM	6 PM
**Guangzhou**			
Unweighted *p*	0.742	0.742	0.742
Weighted *p*	0.999	0.999	0.999
$\gamma _t$	0.652	0.604	0.610
$\alpha$	0.005		
**Shanghai**			
Unweighted *p*	0.956	0.998	0.956
Weighted *p*	0.999	0.999	0.998
$\gamma _t$	0.634	0.604	0.616
$\alpha$	0.004		
**Shenzhen**			
Unweighted *p*	0.956	0.999	0.999
Weighted *p*	0.999	0.999	0.999
$\gamma _t$	0.662	0.612	0.626
$\alpha$	0.005		

## Discussion

By inspecting the structure of those simulated MCNs, we observe that there is a lack of high degree nodes. This observation is confirmed by the generation model, which can be decomposed as the weighted combination of Poisson PDFs as shown in Eq. (5). We see that the degree of a node may be drawn from a collection of Poisson distributions with mean $Mw_i$. This explains the lack of high degree nodes in the contact network as compared to the scale-free network with the same number of nodes and average degree, since the probability of having large *k* in a random network diminishes faster than exponential. On the other hand, the generation model also explains why the tails of the degree distributions of MCNs decay slower than a random network. Note that in MCNs, high degree nodes are generated from the Poisson distribution with a large $Mw_i$ value, which requires a longer trip length $t_i$. The decay of the tails is therefore the convolution of the tail of a random network and the tail of the mobility distribution which decays exponentially. In addition, we derive the expressions for the mean $\langle k\rangle$ and variance ($\sigma ^2=\langle k^2\rangle -\langle k\rangle ^2$) of the MCNs’ degree distributions in SI Appendix S6. We find that $\langle k\rangle \propto N$ and the variance $\sigma ^2\propto N^2$, so that both measures diverge with $N\rightarrow \infty$ and the variance is of higher magnitude than the average node degree. This indicates that the degree distributions of MCNs have similar characteristics as compared to the exponential distribution and the number of contacts and the contact duration are bounded by the human mobility in the transit networks. Moreover, these findings are aligned with the empirical observations of $\langle k\rangle$ and $\langle k^2\rangle$ in simulated MCNs (Fig. S6), which further strengthens the validity of the developed generation mechanism.

One important parameter in the generation model is $\gamma _t$, where we define $\gamma _t$ as the similarity among the trips. To validate this argument, we also quantitatively measure the trip similarity (see SI Appendix S5.2) among travelers based on the trip OD matrix *Q*. The similarity measure is introduced to quantify the strength of overlapping of a particular trip pair on other trip pairs in terms of contact duration and demand level. We then compare the dominant eigenvalues of *Q* and we use the variance of the dominant eigenvalues to quantify the similarity of trip purposes. In particular, a higher variance suggests that most trips are distributed across a few ODs and the trip purposes among these riders are more similar. We compare the computed similarity index with the calibrated $\gamma _t$ and the results are shown in Fig. 5. We see that the calculated similarity presents a strong linear relationship with $\gamma _t$ among all three cities, with $R^2$ value being above 0.77 if we fit a simple linear function to interpret this relationship. These suggest that similarity can be used as a proxy for $\gamma _t$ for prediction purposes.

While it is difficult to devise effective yet practical control strategies, the degree distribution provides valuable insights in improving the resilience of the transit system by controlling how its contact networks are shaped. To reduce the risk of MCNs, it is equivalent to minimize the probability of the MCNs having high degree nodes. Based on Eq. (5), we know that *p*(*k*) is linearly proportional to the number of passengers and the scale of the metro network. Reducing these values will lead to a linear reduction in the average number of contacts while the shape of the degree distribution will remain the same. By observing the metro network layouts, we observe that a larger transit network, possibly with more number of lines and transfer stations, may result in lower $\alpha$. But the data used in our study is not sufficient to explain how we may reduce $\alpha$ and this may require further investigation. Alternatively, efforts can be made to reduce $\gamma _t$ so as to sub-linearly decrease the probability of having high degree nodes and result in the degree distribution that decays faster. This can be achieved by segregating passengers through an optimally designed timetable or advising passengers to distribute their departure time. The ultimate solution, however, lies in the distribution of the human mobility distribution for $t_i$. This will not only reshape how the Poisson PDFs are combined but also change the weight of each Poisson distribution. In particular, we would like to pursue the distributions of $t_i$ with faster decaying tails, so that both $Mw_i$ and $p(w_i)$ for larger $w_i$ values will be minimized at the same time. And this can be realized by changing the layout of the metro network or, ultimately, the urban form itself. We can expect that $w_i$ will decay faster by reducing the number of transfers required for the pair of stations of long trip duration, which results in lower maximum trip length and may also contribute to lowering $\alpha$. As for the urban form, a more decentralized urban structure is the most effective way for reducing the risk of communicable diseases, which implies that people can avoid long distance travels across the city as they can find work or entertainment places closer to their home locations. While both approaches are deemed to be effective, the design of the metro network and urban form is not a sole function of the risk of communicable disease. Thus oftentimes we have to compromise among the disease risk, construction cost, efficiency, and also equity of urban mobility. But the developed model in this study provides an important tool for improving the network resilience without undermining other aspects of the system.

**Figure 6:**
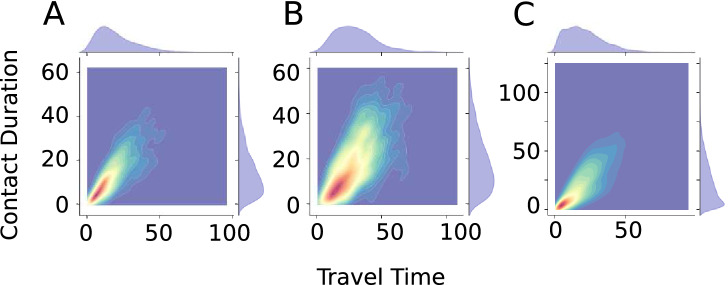
The correlations between travel time and total contagion duration in MCNs of three cities. 50 MCNs are generated for each plot using data from 8:00 to 8:30 AM, with each MCN having 1000 nodes. (**A**)–(**C**) represent results for Guangzhou, Shanghai, and Shenzhen, respectively.

One final issue is to identify the group of travelers who experience and introduce high-risk exposure in the transit system. To gain insights on this issue, the correlations among the travel time distributions and distributions of contact durations are plotted and shown in Fig. 6. We see that the travel time and the contact duration are positively correlated and this observation is consistent across all three cities. We can also verify that there is a wide range of travel time for travelers who experience high contact duration in the metro system. In general, the positive correlation suggests that travelers who experience the highest contact duration are likely to be those who have the longest travel time. And the travel time of urban commuters is closely related to their work and home locations, their income levels, and eventually their lifestyle and health conditions. One recent study reported that those commuters with the longest travel time in the metro are likely to be low-income migrates, and they may change their home and work locations more frequently than other urban commuters^45^. This finding implies another potential risk in transit networks. If commuters with long travel time overlap with the low-income population, then these people are likely to be more prone to infection during disease outbreaks. Compared to other population groups, low-income people usually have fewer options (such as time off and sick leaves) and may pay less attention to personal health and hygiene due to limited disposable income^46^. Consequently, the riskiest group of travelers in metro systems are likely to be the most susceptible and vulnerable group of people during the disease outbreak. And this may inevitably raise additional challenges associated with disease contagion and equity of travel in urban transportation networks.

## Methods

### Unweighted metro contact network

Based on the smart card transaction data and operation data for metro networks, we next develop the algorithm for constructing the MCN. The contact network is constructed at the individual level and we consider both unweighted and weighted contact networks. For unweighted MCN, each node represents a traveler and the link between a pair of nodes denotes that the two travelers will have positive probability to be on the same metro train. Since the smart card data only contain the time and location of a traveler entering and leaving the metro system, we need to infer if two travelers will be on the same metro train based on their trip starting time, origin station and destination station. And this further requires us to predict their travel route within the metro system. While the operation timetable of metro system is largely reliable, we assume that all travelers will follow the shortest route between two trip origin and destination (including both station-wise travel time and transfer time). Based on the predicted travel route, we can therefore determine if a link exists between two travelers following

1. Find the shortest travel route $P_i$ for each traveler.
2. For each pair of passenger *i* and *j*, determine if they have overlapping travel segments $L_{ij}$.
3. If $|L_{ij}|\ge 2$, determine their first meeting location, station *m*, and calculate their arrival time at the meeting station $t_{i,m},t_{j,m}$ respectively.
4. Compute the probability of contact between travelers *i* and *j* based on $t_{i,m},t_{j,m}$ and the frequency *f* of metro lines.
5. Repeat this process until all pairs of travelers are processed. Output *G*.

In step 1, the shortest route can be computed using the Dijkstra algorithm^47^ with the travel time adjacency matrix $\Gamma$, and the shortest routes are stored as sequences of the stations $P_i=\{s_1,s_2,..,s_P\}$ along the routes. In steps 2 and 3, the overlapping travel segments of two travelers *i* and *j* can be identified as the longest common subsequence (LCS) of their routes $p_i$ and $p_j$. In our case, a valid LCS that may grant contact is the LCS of length 2 or higher, indicating that the two travelers share at least one trip segment. In step 4, $t_{i,m}, t_{j,m}$ can be computed from their departure time and the trip time between their origin station and first meeting station *m*. Then their contact probability $p_{ij}$ follows

6 $$\begin{aligned} p_{ij}={\left\{ \begin{array}{ll} 1-\frac{|t_{i,m}-t_{j,m}|}{\frac{1}{f}}, \text {if}\ |t_{i,m}-t_{j,m}|<\frac{1}{f}\\ 0,\,\text {if}\ |t_{i,m}-t_{j,m}|>=\frac{1}{f} \end{array}\right. } \end{aligned}$$

This suggests that two travelers will have positive contact probability if the gap between their arrival time at *m* is less than the headway $\frac{1}{f}$ of metro trains. And this probability decreases linearly considering the uniform arrival of metro trains following frequency *f*. Following Eq. (6), a link will exist in the unweighted MCN if and only if $p_{ij}>0$.

### Weighted metro contact network

Based on unweighted MCNs, we further assign the weight to each link in unweighted MCNs to produce weighted MCNs. In the context of modeling the spread of communicable diseases, the weight on each link has the physical meaning as the *expected contact duration between two individuals within effective transmission range*. By effective transmission range, we consider that two individuals are close enough so that the airborne transmission of a communicable disease is feasible. This follows from the definition of the effective range for droplet transmission, which is usually less than 3 feet while certain diseases such as SARS may reach 6 feet^41^. Let *Z* denote the scaling parameter for the effective transmission range, we have the weight between traveler *i* and *j* as:

7 $$\begin{aligned} d_{ij}=\frac{p_{ij}L_{ij}}{Z} \end{aligned}$$

Considering that a metro train consists of 6 coaches, with each coach of length 72 feet, then Z may take the value of 144 if the effective transmission range is 3 feet. And Eq. (7) characterizes the expected contact duration as the product of the probability for being in effective transmission range $p_{ij}/Z$ and the duration of the contact $L_{ij}$, with the underlying assumption that travelers will uniformly distribute themselves among all metro coaches. The use of *Z* naturally captures the behavior of travelers to avoid congested coaches during travel. With an increasing number of travelers (e.g., more number of nodes in MCNs), this also characterizes the linearly increasing chance of close contacts, where the total contact duration of each individual is the row sum of the contact duration matrix.

Finally, for the transmission rate of communicable disease, let $\beta$ denote the transmission strength per unit time, we have the transmission rate between two travelers as:

8 $$\begin{aligned} \beta _{ij}=\beta d_{ij} \end{aligned}$$

With the above processes, we can use the smart card data to generate sample unweigted and weighted MCNs. Specifically, the smart card data can be aggregated to generate the distributions for trip origin and destinations and the arrival time at each station. We then sample *N* travelers following the distributions, where each traveler has their time of arrival, the origin station and the destination station. And the MCNs with *N* nodes can consequently be constructed following the generation process for MCNs. We denote *A* as the adjacency matrix of the generated MCNs, with each entry $A_{ij}=d_{ij}$.

### Ethical statement

The anonymous smart card transaction data is obtained from collaborations in China with our partners and all required permissions were obtained by them for research use. The data that is used in this research does not identify any human subjects and does not provide any identifiers of individual specific information, and is therefore exempt from any IRB approvals. All authors of the paper had no access to identifying information when analysing the data. In addition, the study is not part of any funded project and permission from the funding agencies is not required.

### Data usage

The usage of the metro smart card transaction data in this study are permitted by Guangzhou Metro, Shanghai Metro and Shenzhen Metro.

## Supplementary Information

Supplementary Information.
