# Supplementary material for: Scaling of contact networks for epidemic spreading in urban transit systems
Source: Sci Rep. 2021 Feb 23;11:4408. doi: 10.1038/s41598-021-83878-7 (PMC7902662; doi:10.1038/s41598-021-83878-7)
Supplement: Supplementary file 1 — Supplementary Information. [file 41598_2021_83878_MOESM1_ESM.pdf]

Supplementary Information

Scaling of contact networks for epidemic spreading in urban transit systems

Xinwu Qian, Lijun Sun, Satish V. Ukkusuri

S1 Data

S1.1 Metro Trip data

The metro smart card transaction data are from three major cities in China: Shanghai, Guangzhou, and Shenzhen. These data have a similar structure, with each record containing the information of smart card ID, transaction ID, transaction time, boarding station/time, and alighting station/time. The transaction type indicates if the transaction is an entry or exit at the transaction station. Since each smart card is associated with a unique ID, we can therefore construct the trip sequences for each commuter (each card) based on the transaction time, transaction type, and location. We present a sample of smart card transaction data of Shenzhen on April 21st, 2016 in Tab S1.

Table S1: Sample records of metro transaction data of Shenzhen (2016-04-21). For Transaction Type, 21 indicates that the traveler left the system and 22 denotes the entry of the traveler.

User ID	Transaction Type	Time	Station ID
80357781	22	08:39:50	1
290452424	22	08:39:32	1
20353676	22	09:41:43	1
361341888	21	07:15:36	1
329838057	22	07:47:08	1
667519928	22	08:34:07	1
329213920	21	07:37:19	1

We have access to three months of data for Guangzhou in 2017, one week of data for Shanghai in 2015, and 8 days of data for Shenzhen in 2016. A summary statistics of the data and the size of the metro networks that corresponded to the period of available data is shown in Table S2. For each city, we extract the data of five consecutive weekdays for further analysis (2017.07.03 - 2017.07.08 for Guangzhou, 2015.04.13 - 2015.04.17 for Shanghai, and 2016.04.14 - 2016.04.15 & 2016.04.18 - 2016.04.20 for Shenzhen). There were no national holidays or major events during the selected time periods. When compared with official statistics of daily ridership, we observe that the smart card transaction data may cover over 60% of total daily travelers and can well reflect the trip dynamics of regular metro users. The metro networks in these cities have distinct layouts which are tailored to the urban form. Shanghai metro is the metro system with the longest total mileage and largest number of stations. It also has the highest number of daily travelers. Guangzhou and Shenzhen are similar in terms of the size of the metro networks, however, the shape of the metro network differs. In particular, Shenzhen is a stripe-shape city where commercial areas are located in the middle and residential places are distributed at east and west sides of the city. The layouts of metro networks and half-hourly passenger demand distributions of the three cities are presented in Figure S1. Note that the layouts presented here correspond to the period of time when the data were collected.

While only five weekdays of data are used for each study, we further demonstrate the representativeness of the selected data. Due to the limited data availability in the other two cities, we here use the selected one week data in Guangzhou and compare it with the metro ridership patterns in the other 12 weeks. In general, the metro system usage tends to be highly regular and this can be seen in Figure S1d. In particular, little variations in half-hourly ridership are observed for both peak periods of the day (7-10 and 17-19). Moreover, while the half-hourly ridership shows higher variation during off-peak hours, the trends of the selected week largely agree with those of the other 12 weeks. Aside from the distributions of half-hourly ridership, the data representativeness can also be seen from statistical metrics. Figure S1e presents the box plot comparison among the 13 weeks, with week 1 being the selected week. We can tell that there are minimal differences in terms of the minimum, maximum and mean values as well as the upper and lower 25-th quantiles. Finally, we also perform the two-sample K-S test for the selected one week

data and the other 12 weeks of data. The null hypothesis is that the selected week of data is drawn from the same distribution as the other data of the other 12 weeks. The test returns a p-value of 0.606, which is significantly higher than the 0.05 level. As a result, we failed to reject the null hypothesis and we shall have high confidence that the selected data are presentative of the regular metro usage patterns.

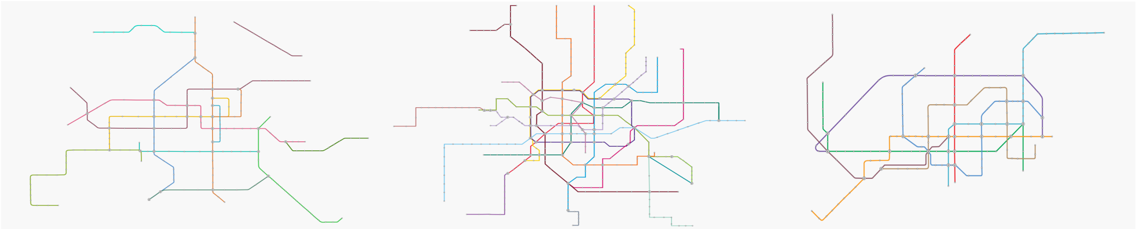

(a) Guangzhou metro network (b) Shanghai metro network (c) Shenzhen metro network

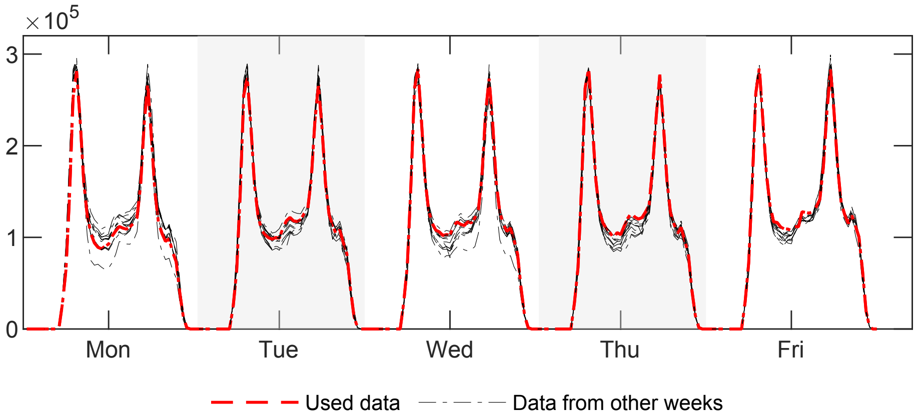

(d) Comparison of Guangzhou metro's half-hourly weekly ridership distribution between the selected week and the metro ridership in other 12 weeks

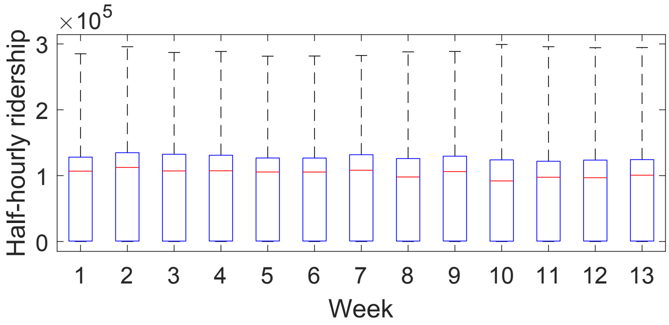

(e) Box plot for comparing the distribution of half-hourly metro ridership among the 13 weeks in Guangzhou. Week 1 represents the selected week for the analyses.

Figure S1: Metro network layouts of the three cities and the half-hourly passenger demand distributions.

Table S2: Summary of metro card transaction data from three major cities in China

City	Start date	End date	# metro lines	# stations	Average daily records
Guangzhou	2017.07.01	2017.09.30	8	166	1.6 million
Shanghai	2015.04.13	2015.04.20	13	288	4.16 million
Shenzhen	2016.04.14	2016.04.21	5	118	2.13 million

S1.2 Operation data

In order to infer the contact among travelers, we also need operation data which include the trip time between two adjacent metro stations, the approximate transfer time at transfer stations, and the frequency of metro trains.

To obtain these data, we developed web crawlers and extracted the metro station adjacency matrix from GaoDe Map API [1] as the representation of the metro system layouts . In addition, the time tables of the three metro systems were obtained from their official websites [2, 3, 4], which contain the travel time between two stations as well as the frequency of the metro trains during different time periods. Finally, the transfer time required at the transfer station is calculated by identifying a route that needs a transfer at the station, quoting the travel time of the route using GaoDe Map API and subtracting the travel time of the route based on the values that we obtained from the timetable.

S2 Metro Contact Network

S2.1 Structural property of MCNs

Table S3: Summary statistics of the MCNs of various number of nodes for Guangzhou. In the table, $< k >$ represents the average unweighted degree and $< d >$ represents the average weighted degree.

Number of nodes	Average path length	Average clustering coefficient	Assortativity	Diameter	$<k>$	$<k^2>$	$<k_{max}>$	$<d>$	$<d^2>$	$<d_{max}>$
500	2.99	0.48	0.27	6.90	18.00	416.37	45.10	7.97	102.52	30.74
1000	2.79	0.49	0.26	7.20	35.09	1,562.10	89.50	15.95	401.66	59.99
1500	2.68	0.49	0.24	6.80	53.64	3,610.10	126.70	23.68	873.08	89.61
2000	2.64	0.49	0.25	7.10	71.03	6,322.30	169.70	31.83	1,564.80	117.92
2500	2.60	0.49	0.25	6.30	89.55	10,027	209.70	40.22	2,503.40	148.33
3000	2.58	0.49	0.25	6.40	106.15	14,085	247.20	47.35	3,434.80	173.65
3500	2.55	0.49	0.25	6.60	124.88	19,444	292.20	56.30	4,862.50	208.17
4000	2.54	0.49	0.24	6.40	141.92	25,075	337.50	63.51	6,168.50	237.69
4500	2.52	0.49	0.26	5.90	160.94	32,346	378.70	72.05	7,966.40	259.52
5000	2.51	0.49	0.24	6.40	177.68	39,327	411.20	79.09	9,583.30	292.39
5500	2.50	0.49	0.25	6.20	197.53	48,585	461.80	88.56	12,000	318.63
6000	2.49	0.49	0.24	5.90	212.67	56,242	491.40	95.82	14,058	354.27
6500	2.49	0.49	0.25	6.20	231.81	67,051	541.50	104.12	16,644	377.04
7000	2.48	0.49	0.25	5.90	249.96	77,836	579.30	112.71	19,554	401.65
7500	2.47	0.49	0.25	5.80	267.74	89,419	626.30	119.68	21,986	443.35
8000	2.46	0.49	0.25	5.90	285.40	101,360	661.60	127.38	24,787	460.05
8500	2.46	0.49	0.23	6.20	302.02	113,320	688.30	134.64	27,515	494.58
9000	2.45	0.49	0.24	5.90	321.19	128,210	738.70	144.12	31,663	520.86
9500	2.45	0.49	0.25	6.20	339.39	143,330	776.80	152.16	35,326	541.30
10000	2.44	0.49	0.25	6.00	356.57	158,060	819.80	159.84	38,966	576.08

We simulate MCNs of different sizes to gain insights into the structural properties. We present the summary statistics of the MCNs of various number of nodes in Table S3 to S5. We are interested in the following representative network metrics and these metrics are the average of 10 random realizations of MCNs:

1. Average path length measures the mean shortest path length among all pair of nodes in MCN.
2. Average local clustering coefficient is calculated following the definition in [5] and measures the average cliquishness of individual travelers.
3. Assortativity measures the proclivity of a node to attach to another node of a similar degree. This metric quantifies the similarity of the nodes that get into contact.
4. Diameter measures the longest shortest path of the MCNs.
5. $< k >$ is the average unweighted degree of the MCNs.

Table S4: Summary statistics of the MCNs of various number of nodes for Shanghai.

Number of nodes	Average path length	Average clustering coefficient	Assortativity	Diameter	$<k>$	$<k^2>$	$<k_{max}>$	$<d>$	$<d^2>$	$<d_{max}>$
500	3.14	0.46	0.27	7.40	14.48	280.46	41.90	9.50	139.37	35.67
1000	2.89	0.46	0.30	7.60	29.14	1125.50	77.20	18.78	528.15	67.91
1500	2.77	0.47	0.29	7.80	43.56	2483.10	117.40	28.37	1179.50	102.35
2000	2.71	0.47	0.30	7.60	58.74	4516.80	154.30	37.91	2085.60	136.65
2500	2.67	0.47	0.30	7.80	73.57	7098.20	192.60	47.48	3275.60	172.57
3000	2.64	0.47	0.30	8.30	87.83	10030	231.50	57.26	4737.90	204.34
3500	2.62	0.47	0.29	7.50	102.58	13642	274.60	66.58	6386	240.23
4000	2.60	0.47	0.30	7.40	116.81	17751	306.90	75.84	8276.20	261.84
4500	2.59	0.47	0.30	7.30	131.69	22497	344.40	85.60	10504	295.58
5000	2.57	0.47	0.30	7.30	146.25	27856	389.10	95.46	13144	335.95
5500	2.56	0.47	0.30	7.40	160.56	33407	423.10	104.59	15692	374.46
6000	2.55	0.47	0.29	7.30	174.79	39561	465	113.71	18553	410.25
6500	2.54	0.47	0.29	7.30	190	46854	504.10	123.32	21845	429.61
7000	2.53	0.47	0.30	7.10	205.03	54479	552.70	133.28	25513	471.11
7500	2.52	0.47	0.29	8	221.12	63337	584	143.07	29267	492.32
8000	2.52	0.47	0.29	8	232.36	69754	609.50	151.18	32655	526.39
8500	2.51	0.47	0.29	6.90	251.55	81864	656.70	164.12	38671	575.84
9000	2.50	0.47	0.29	7.20	263.96	89996	698	171.97	42270	612.77
9500	2.50	0.47	0.29	7.40	277.66	99696	734.20	180.60	46780	641.35
10000	2.49	0.47	0.30	7.10	292.74	110970	778.80	190.79	51981	676.15

Table S5: Summary statistics of the MCNs of various number of nodes for Shenzhen.

Number of nodes	Average path length	Average clustering coefficient	Assortativity	Diameter	$<k>$	$<k^2>$	$<k_{max}>$	$<d>$	$<d^2>$	$<d_{max}>$
500	3.01	0.53	0.26	6.70	20.51	552.23	51.60	11.54	216.49	42.15
1000	2.78	0.54	0.27	6	41.59	2227.10	101.20	23.45	874.57	86.62
1500	2.69	0.54	0.25	5.20	61.70	4882.30	154.60	35.44	1996.70	132.55
2000	2.64	0.54	0.23	5.60	83.32	8839	199.90	47.70	3570.50	175.15
2500	2.60	0.54	0.26	5.10	104.38	13918	272.10	60.10	5650.10	223.27
3000	2.57	0.54	0.25	5.10	125.54	20095	328.70	71.94	8064	256.44
3500	2.56	0.54	0.24	5	144.51	26587	361.90	82.46	10620	295.50
4000	2.55	0.54	0.24	5.10	166.57	35318	404.40	95.47	14250	354.75
4500	2.53	0.54	0.24	5	187.63	44732	466.60	107.36	17976	390.15
5000	2.53	0.54	0.25	5	206.49	54322	522.20	118.88	22117	437.16
5500	2.51	0.54	0.24	5	228.79	66332	582.80	131.54	26888	491.68
6000	2.50	0.54	0.24	5	249.88	79360	656.50	143.23	31951	518.46
6500	2.50	0.54	0.24	4.80	269.98	92430	728.10	154.43	37126	571.30
7000	2.49	0.54	0.24	5.20	291.62	108070	776.50	167.16	43457	621.85
7500	2.48	0.54	0.24	5	311.65	123240	831.90	177.91	49027	641.73
8000	2.48	0.54	0.24	5	333.22	141000	893.10	190.60	56325	699.74
8500	2.48	0.54	0.24	4.80	354.44	159240	942.40	203.12	63857	724.36
9000	2.47	0.54	0.24	4.80	372.76	176220	980	213.35	70824	769.69
9500	2.47	0.54	0.23	4.80	395.30	198090	997.20	225.65	78760	792.65
10000	2.46	0.54	0.24	4.80	414.51	217520	1068.70	237.12	87273	871.27

6. $< k^2 >$ is the average second moment of the unweighted degree of the MCNs.
7. $< k_{max} >$ is the maximum degree of the unweighted MCNs.
8. $< d >$ is the average weighted degree of the MCNs.
9. $< d^2 >$ is the average second moment of the weighted degree of the MCNs.
10. $< d_{max} >$ is the maximum weighted degree of the MCNs.

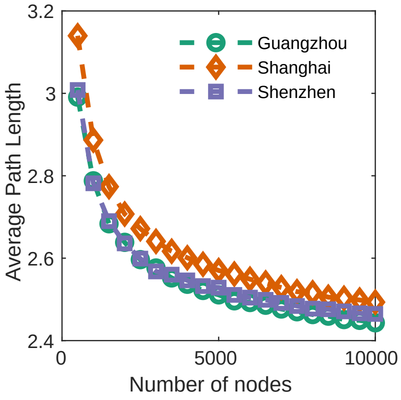

Figure S2: Change of average path length with increasing number of nodes in MCNs.

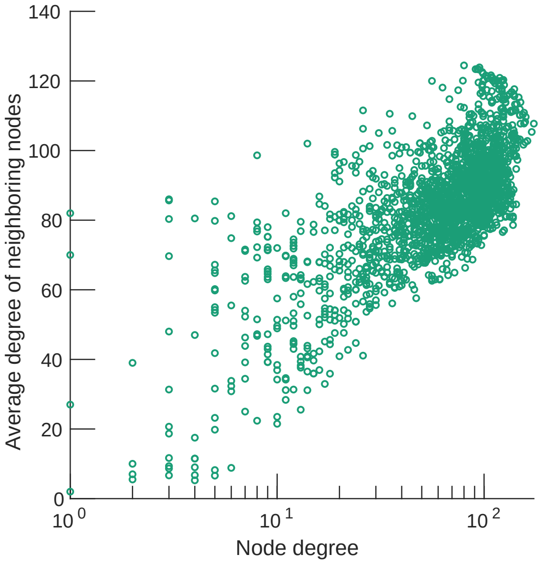

Figure S3: Correlation between node degree and the average degree of neighboring nodes. The results are obtained from a sample MCN with 1000 nodes for Guangzhou during 8:00-8:30 AM.

Despite the differences in scale and layout of the metro networks, we can immediately observe several structure properties that are universal across the MCNs. The MCNs of different cities and a various number of nodes all present high values of average local clustering coefficient, short average path lengths and small network diameters. Moreover, these statistics are found to converge to fixed values with the number of nodes increases from 500 to 7,000 and then become invariant with further increases in the number of nodes in the network (see Fig. S2 for the convergence of average path length). These results suggest that the structural properties of the MCNs are primarily determined by the layout and scale of the metro network. And the minor differences in the values of these network metrics are also reflections of the differences in their metro systems. Since Shanghai has the largest metro network, we observe the average path length and the network diameter are in general higher than those of Guangzhou and Shenzhen, and the average local clustering coefficient is comparatively lower than other cities due to more diverse destinations among travelers. Finally, the assortativity values of the three cities imply that MCNs are weakly assortative where nodes are likely to be connected to other nodes with a similar degree and this can be verified from the visualization

in Fig. S3. In general we observe a positive correlation between the node degree and the average degree of the neighboring nodes, but there is also a huge discrepancy among the average degree of the neighboring nodes for the nodes of similar degree. This indicates a certain level of randomness in the number of contacts in the MCN, which is likely to depend on the time of arrival and the specific pair of trip origin and destination.

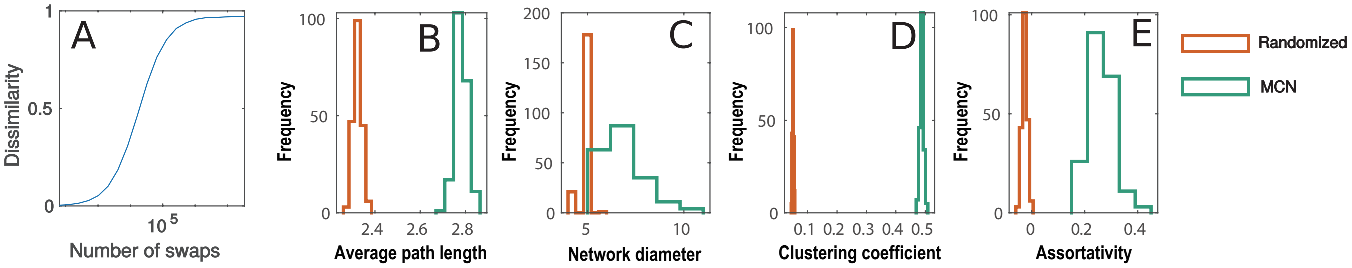

Figure S4: Randomization of simulated MCNs with 1000 nodes using data from Guangzhou during 8:00 to 8:30 AM. (A) presents the dissimilarity between the randomized network and the original MCN with increasing number of swaps. The two networks become almost completely dissimilar with 2^{22} swaps. We compare the distribution of the average path length (B), network diameter (C), average local clustering coefficient (D) and assortativity (E) before and after the randomization using 20 samples of the simulated MCNs.

S2.2 Network randomization

To verify the statistical significance of the network metrics for MCNs, we conduct the randomization of the simulated MCNs by selecting two random links in the MCN and swap their endpoints, which is also known as XSwap [6]. This process creates a completely randomized counterpart of the generated MCN, and we can then compare their structural properties to validate if the structure characteristics discussed above specifically pertain to the MCNs. We note that XSwap produces the randomization of the MCN while preserving the same degree distribution. We compare the network metrics discussed above among 200 samples of MCN and the corresponding randomized networks, and the results are shown in Fig. S4. It is obvious that the distributions for MCNs and the random counterparts are independent for all the metrics.differences in the distributions of network metrics between the two networks are statistically significant. And we further conduct the two-sample t-test for these metrics, with the null hypothesis being that the sample metrics from MCNs and the randomized networks are from the distribution with the same mean and variance. The results reject the null hypotheses with p-values closing to 0. This confirms the observed structural properties are distinct in MCNs. These results highlight that MCN is a special type of network that presents universal structural properties though being stemmed from metro systems of different scales and layouts.

S2.3 Degree distribution

The universality of the MCN can also be observed from its unweighted and weighted degree distributions. We first observe that with the same number of nodes in MCNs, the average unweighted degree and weighted degree are different among the three cities. Shenzhen metro has the highest average unweighted and weighted node degree and also the largest variation of node degree, followed by Guangzhou and Shanghai respectively. This is likely because that Shenzhen has the smallest metro network among the three which results in higher chance of contact and hence higher clustering coefficient and average node degree. But the probability density functions for the degree distributions of the three cities present striking similarities. As shown in Fig. S5, the unweighted degree distribution shows that there is a large proportion of nodes of degree smaller or equal to around half of the maximum degree in MCN and it has a tail that decays almost exponentially fast. One may be tempted to fit a power-law distribution to explain the decay of the tail. Indeed, many real networks are observed to be well explained by power-law distribution and we observe similar decaying trend between MCN and the power-law counterpart. And the MCNs are shown to be significantly different from the random networks in terms of the overall shape and the tail behavior. But there are two subtle differences that prevent the use of power-law distribution for characterizing the degree distribution of MCNs. First, as seen in Fig. S5, the chance of having high-degree hubs in MCN is much lower as compared to the scale-free network of the same number of nodes and links. This indicates the decay of the tail is faster than that in the scale-free network. But more importantly is that MCNs are deemed to be scale-dependent and the degree distribution is closely associated with the number of nodes or equivalently the number of travelers in the metro system. This poses a fundamental contradiction to the philosophy behind the power-law distribution and its properties.

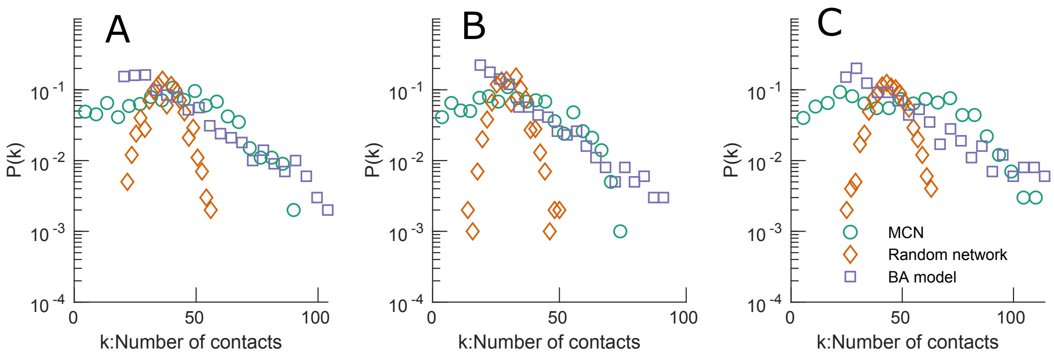

Figure S5: Comparing the probability density function of the degree distribution of MCNs with the random networks and scale-free networks (generated using the Barabasi–Albert (BA) model) with same number of nodes (N=1000) and links for (A) Guangzhou, (B) Shanghai and (C) Shenzhen.

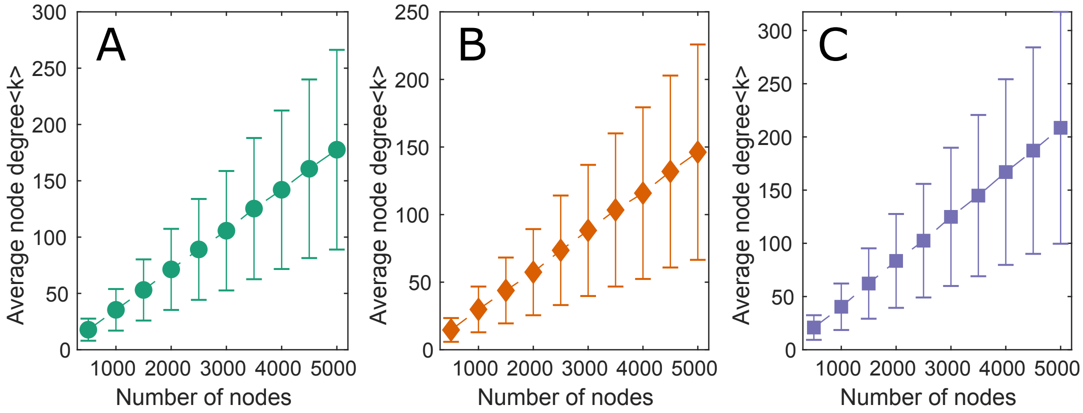

Figure S6: Change of node degree and the standard deviation of node degree with increasing number of nodes in MCNs. (A) Guangzhou, (B) Shanghai and (C) Shenzhen.

Nevertheless, the degree distribution of MCN presents several surprising properties that are usually seen in the scale-free network. The first is the possible divergence of $< k^2 >$ and $< d^2 >$ as shown in Fig. S6, where the standard deviation of the node degree increases with higher average node degree. Such a phenomenon is one important reason that leads to the presence of scale-free property and this is observed in the MCNs for all three cities. In addition, despite seeing that the chance of large hubs is much lower in MCN than in the scale-free network, we empirically observe that the maximum degree of the MCN also increases linearly with an increasing size of the network. This again is a unique property that is found in scale-free networks:

$$k_{max} \propto N^{\frac{1}{\gamma-1}} \tag{1}$$

where γ_t is the exponent the power-law distribution.

In summary, by analyzing the simulated MCNs, we find that several structural properties of the MCNs are invariant to the size of the network and are primarily determined by the layout and scale of the metro systems. We show that these properties are rare in random networks and are likely to be distinct features of MCNs that are universal across different cities. But more importantly, while presenting fundamental differences when compared with scale-free networks, the MCNs also present universal structural properties that are usually found in scale-free networks. These findings define the MCN as a special class of networks that arises from the collective behavior of travelers and also the interplay between trip patterns and the metro system layout.

S3 Individual level disease transmission model

Based on the constructed MCNs, we next model the percolation of communicable diseases on the MCNs with the individual based model (IBM). The IBM is adapted from the non-linear dynamical system approach in [7]. In the IBM, each traveler is a node in MCN and the transmission takes place between two travelers with positive β_{ij}. We consider the classical susceptible-infectious-susceptible (SIS) model as the disease dynamics, while a more refined model such as SIR and SEIR can also be embedded.

The IBM model takes the following items as model input:

1. The unweighted adjacency matrix G or weighted adjacency matrix A of the MCN.
2. Disease parameters: unit transmission rate β and recovery rate r, where $1/r$ represents the unit number of time steps required for a full recovery.

S3.1 Disease transmission rate

For communicable diseases that spread upon contact, it is well understood that the exposure duration and contact distance between two individuals are two contributing factors to successful transmission. In our study, the strength of transmission between two individuals is measured by the expected contact duration of two travelers based on their travel profile, and scales the probability of contact by considering the chance if two individuals are within effective transmission distance. As a consequence, we are able to measure the heterogeneous transmission rate between two travelers.

S3.2 The model

Denote $p_{i,t}$ as the probability that node i is infected at time t. When an individual i travels, the probability that i stays healthy at time t can be written as:

$$1 - p_{i,t} = (1 - p_{i,t-1})q_{i,t} + p_{i,t-1}r \tag{2}$$

where $q_{i,t}$ represents the probability that neighboring nodes of i fail to transmit disease to node i at time t. The first term on right hand side of the equation implies the node was healthy at time $t-1$ and is not infected at time t, and the second term suggests that the node was infected at time $t-1$ but recovered at time t.

The probability that all neighbors of i failed to transmit the disease can be written as:

$$q_{i,t} = \prod_{j \in \mathcal{N}(i)} (1 - p_{j,t} + (1 - \beta_{i,j})p_{j,t}) \tag{3}$$

with $\mathcal{N}(i)$ denotes the set of neighboring nodes of i. The right hand side also also contains two parts: either a neighbor j is not infectious at current time t $(1 - p_{j,t})$, or if j is infectious but fails to transmit the diseases.

By rearranging equation 2, we can express the probability that node i is infected at time t as

$$p_{i,t} = 1 + p_{i,t-1}(q_{i,t} - r) - q_{i,t}, \forall\, i \in V \tag{4}$$

And the entire system dynamics over the MCN can be expressed in the matrix form as

$$\mathbf{P}_t = \mathcal{G}(\mathbf{P}_{t-1}) \tag{5}$$

So that the disease spreading on MCN is characterized as a non-linear dynamic system.

S3.3 Condition for disease free equilibrium

The disease dynamic system on MCN has two equilibrium states. One is the disease free equilibrium (DFE), where each individual is in S (healthy) state and the disease is completely eliminated. On the contrary is the endemic equilibrium, where there will always be a positive portion of nodes that are in I state. Formally, the DFE can be defined as

Definition 1 (Disease free equilibrium (DFE))**.** *The system reaches the disease free equilibrium if at time t* $p_{i,t} = 0$ *for all nodes.*

The vulnerability of a metro system therefore corresponds to the stability condition for the IBM of the MCN to reach DFE. The stability of the system depends on how the system may return to equilibrium under small perturbation. If the perturbation diminishes and the system goes back to the equilibrium point, the DFE point is said to be asymptotically stable, otherwise the system will reach the endemic state. Before we establish the stability condition for DFE, we first introduce the Gershgorin Circle theorem [8] as follows

Theorem 1 (Gershgorin circle theorem)**.** *Every eigenvalue of a complete matrix A lies within at least one of the Gershgorin discs* $D(a_{i,i}, R_i)$*:*

$$|\lambda - a_{i,i}| = \left|\sum_{j \neq i} a_{i,j}x_j\right| \leq \sum_{j \neq i} |a_{i,j}||x_j| \leq \sum_{j \neq i} |a_{i,j}| = R_i. \tag{6}$$

where λ *is the eigenvalue of A.*

Based on Gershgorin circle theorem, we develop the following condition for the stability of the DFE on MCN:

Proposition 1. *The DFE is asymptotically stable if* $max_i(\sum_j(\beta_{i,j})) < r$.

Proof. We proof the proposition by linearizing the non-linear dynamic system $\mathcal{G}(P_{t-1} = 0)$ at the DFE and measuring the partial derivatives K:

$$K = \frac{\partial \mathbf{P}_t(0)}{\partial \mathbf{p}_{t-1}} \tag{7}$$

where we have

$$K_{i,j} = -r + 1, \quad \text{if } i = j \tag{8}$$

$$K_{i,j} = \beta_{i,j}, \quad \text{if i}\neq\text{j and i,j are adjacent} \tag{9}$$

$$K_{i,j} = 0, \quad \text{o.w.} \tag{10}$$

Therefore we have

$$K = (1 - r)I + B \tag{11}$$

For the DFE to be stable, it must be satisfied that the largest eigenvalue of K is less than 1:

$$\rho(K) < 1 \tag{12}$$

Define δ as an upper bound of the eigenvalue of K. Since all diagonal entries of K are identical, by applying Theorem 1, we have

$$\rho(K) \leq \delta = \max_i(R_i(B)) + K_{i,i} = \max_i(\sum_j(\beta_{i,j})) + 1 - r \tag{13}$$

To satisfy the condition in equation 12, we require the upper bound δ to satisfy:

$$\max_i(\sum_j(\beta_{i,j})) + 1 - r < 1 \tag{14}$$

This gives that $max_i(\sum_j(\beta_{i,j})) < r$ and completes the proof. □

Proposition 1 has several important implications. The risk level of the MCN is shown to be dictated by the individual who has the highest risk exposure. As long as the exposure rate of this particular individual is smaller than the recovery speed, the system will reach DFE. Otherwise the system may be either DFE or endemic. However, in practice, if we would like to control the spread of communicable diseases, it is unlikely that we may identify who exactly this person is. Even if this person is spotted, vaccine/quarantine the individual does not necessarily reduce the risk level of the overall system, since the second riskiest person may have a similar level of risk exposure. This implies that we would also need to examine the structure of the contact network to devise feasible control strategies. In addition, the model provides the solution to monitor the vulnerability of metro systems at very fine scale and identify the periods of time that are of particularly high risk level.

S4 OD-level model

One drawback of the IBM model, however, comes from its computational bottleneck. It will be very expensive to generate the large-scale MCN with millions of passengers, which copes with the passenger demand level in real-world scenarios. In this regard, we also develop a metapopulation model based on the flow of travelers between each pair of origin and destination (OD) pair. The OD level model can be used to monitor the risk level of metro systems, but it does not reveal any insights on the contact pattern among individual travelers. The OD-level model treats each pair of OD as the set of nodes and the contagion pattern between OD pairs as the set of links. It can be readily seen that the total number of OD pairs in a given metro network is the square of the number of stations, which is much more scalable as compared to constructing contact networks for millions of travelers. Denote S_i and I_i as the susceptible population and infected population of OD pair i, and let $\mathcal{P}$ be the set of OD pairs in the network, we have the following equations

$$E_{i,j} = \beta \bar{d}_{j,i} S_i I_j \tag{15}$$

where $E_{i,j}$ represents the proportion of susceptible population of i being infected by the infectious population of j and $\bar{d}_{j,i}$ is the expected contact duration between OD pairs i and j. The disease dynamics at the OD level can therefore be written as:

$$\frac{dI_i}{dt} = -rI_i + \sum_{j\in\mathcal{P}} E_{i,j}, \forall i \in \mathcal{P} \tag{16}$$

Moreover, since $I_i + S_i = N_i$, where N_i is the total number of travelers for OD pair i, equation 16 can be further rewritten as:

$$\frac{dI_i}{dt} = -rI_i + \sum_{j\in\mathcal{P}} \beta \bar{d}_{j,i} N_i I_j - \sum_{j\in\mathcal{P}} \beta \bar{d}_{j,i} I_i I_j, \forall i \in \mathcal{P} \tag{17}$$

And the matrix form is therefore

$$\frac{dI}{dt} = FI + b(I) \tag{18}$$

where F is a square matrix with its entry: $F_{ii} = \beta \bar{d}_{i,i} N_i I_i - r$ and $F_{ij} = \beta \bar{d}_{j,i} N_i I_j$. $b(I)$ is a column vector with its entry being $b(I)_i = -\sum_{j\in\mathcal{P}} \beta \bar{d}_{j,i} I_i I_j$. Equation 18 gives the disease dynamics at the OD level.

We can see that one important difference between the OD-level model and the IBM is that the OD-level model use $\bar{d}_{ij}$ as the aggregate representation of the contact duration between all travelers of OD pair i and travelers of OD pair j, rather than the individual level contact duration d_{ij} between travelers i and j. As a result, it sacrifices the fidelity for modeling disease at the individual model, but can be used for understanding the system level dynamics more efficiently.

S5 Generation model

By observing that multiple metro networks in different cities share very similar degree distributions in their MCNs, we next establish the generation mechanism to model how the MCNs are shaped during travel. The goal is to build a single generation mechanism that is capable of restoring the MCNs of all cities to support the universality of MCNs.

Being different from many other networks, the MCNs are special in the way that **the number of links each node being adjacent should be a function of the total number of nodes in the MCNs.** This corresponds to the congestion effect in the metro system with more number of travelers. Consequently, the MCNs may not be generated in a way like preferential attachment [9] where new nodes and links are added sequentially. Instead, we follow a process where we first estimate the total number of links in the network and then assign the links among the nodes in a way similar to the configuration model.

To cope with the congestion effect in MCNs, we consider the expected number of contacts each node may encounter as:

$$c_i = \alpha t_i^{\gamma_t} (N-1) \tag{19}$$

This states that the number of contacts is proportional to the rescaled travel time $t_i^{\gamma_t}$ and the number of nodes N. In particular, with $0 < \gamma_t \leq 1$, $t_i^{\gamma_t}$ suggests more expected number of contacts with increasing travel time, and if we take the derivative of $t_i^{\gamma_t}$ with respect to t_i, we have

$$\frac{dt_i^{\gamma}}{dt_i} = \gamma_t t_i^{\gamma_t - 1} \tag{20}$$

which implies that, in contrast to the rescale as γt_i, the number of contacts does not increase linearly with increasing t_i. Instead, the increase rate will drop with the increase in travel time. Indeed, the number of contacts one may have with 40 minutes of travel should not be 10 times that of the number contacts as if one travels for 4 minutes. Regarding the value of γ_t, we define it as the similarity coefficient that measures the 'similarity' of travels among all travelers. A higher value of γ_t indicates that, on average, a traveler will have a higher contact chance with another traveler, e.g., two travelers are more likely to travel in the same direction to the same destination. On the other hand, the value of α reflects the scale of the metro network, with the physical meaning being the contact rate per individual traveler per unit time of travel. As a result, α is a system dependent value and varies across the cities.

With the above definition, we can approximate the total number of links (contacts) in the MCNs as:

$$C = \sum_{i=1}^{N} c_i \tag{21}$$

And if we consider each link as two stubs (half links), denoting $M = 2C$ and $m_i = 2c_i$, we then assign these stubs to each node based on their contribution $t_i^{\gamma_t}$, where the probability that a randomly chosen stub is adjacent to node i with travel time t_i as:

$$w_i = \frac{m_i}{M} = \frac{t_i^{\gamma_t}}{\sum_{j=1}^{N} t_j^{\gamma_t}} \tag{22}$$

While each stub counts as one degree for each node, we can therefore write down the probability density function that a node of travel time t_i is of degree k follows the binomial distribution:

$$p(k|t_i) = \binom{M}{k} w_i^k (1-w_i)^{M-k} \tag{23}$$

Then for a randomly selected node in the MCN, the probability density function for the number of contacts follows

$$p(k) = \sum_{i=1}^{N} p(t_i) \binom{M}{k} w_i^k (1 - w_i)^{M-k} \tag{24}$$

where $p(t_i)$ is the probability density function for the human mobility within metro system and is observed to be well captured by the exponential distribution. With large M, we can approximate the binomial distribution as the Poisson distribution and hence we have

$$p(k) = \sum_{i=1}^{N} \frac{(Mw_i)^k e^{-Mw_i}}{k!} p(t_i) \tag{25}$$

and this gives the probability density function for the unweighted MCN.

Following equation 25, we can subsequently generate a MCN with M stubs attached to each node. To produce the weighted MCN, we follow the process of the configuration model as:

1. Randomly selected two stubs in the unweighted MCN, with the nodes adjacent to the stubs as i and j.
2. Connect the selected stubs with an link, and assign the weight to the link: $d_{ij} \propto min(t_i, t_j)$.
3. Repeat the above two steps until all stubs are exhausted. Output the weighted MCN.

In summary, the above procedure describes the growth of MCN as a two-stage process where we first determine if two travelers will get into contact and then decide the duration of their contact which is assumed to be proportional to the shorter travel time of the two.

S5.1 Validation

The validation of the generation model involves the calibration of the model parameters and then verify if the calibrated generation model is representative of the simulated MCNs from the smart card data. The correctness of the generation model is validated using the two-sample Kolmogorov–Smirnov (KS) test [10] to compare the CDF of the degree distribution of the MCN from the generation model and the CDF of the degree distribution of the MCNs simulated from the smart card data. The null hypothesis of the KS test is that the two data samples for comparison are drawn from the same continuous distribution. Specifically, we bin the degree distribution of each MCN into 20 equal-length intervals and conduct KS test on the probability distribution of the binned data.

The calibration of model parameters is to find the best γ_t and α value that leads to the best goodness of fit between the generation model and the simulated MCNs. In particular, we have different γ_t for different time intervals while α is held the same across all time intervals for a particular city. Since we do not have a closed-form representation for the probability density function of the weighted MCNs, we conduct cross-validation to find the pair of parameters that minimizes the KS statistics. For each city, we consider α being time-invariant since it captures the impacts of metro network structure, and γ_t will change over time to reflect temporal variations of passenger trip patterns. We perform cross-validation to determine the optimal α and γ_t for each city and for each time period, with the selection criteria being the parameter combination that gives the lowest sum of KS statistics for weighted degree distribution and unweighted degree distribution of the MCNs.

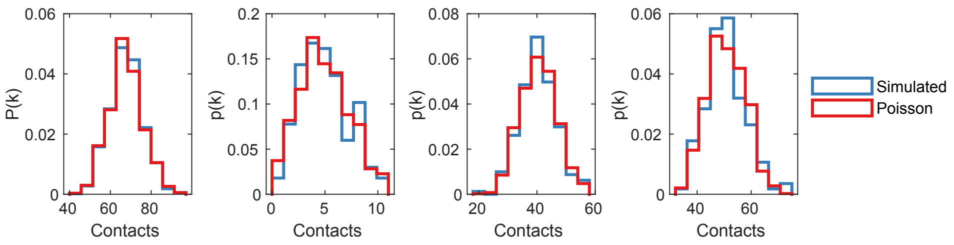

Figure S7: Validation of the Poisson distributed number of contacts.

S5.2 Measure of similarity

To further validate the correctness of γ_t, we present the metric for measuring the trip similarity among all travelers and we compare the computed metrics to the fitted γ_t values. The similarity is measured by first constructing the correlation matrix Q of the trip pairs, where for each entry of Q:

$$Q_{i,j} = f_i f_j \sigma_{i,j} \tag{26}$$

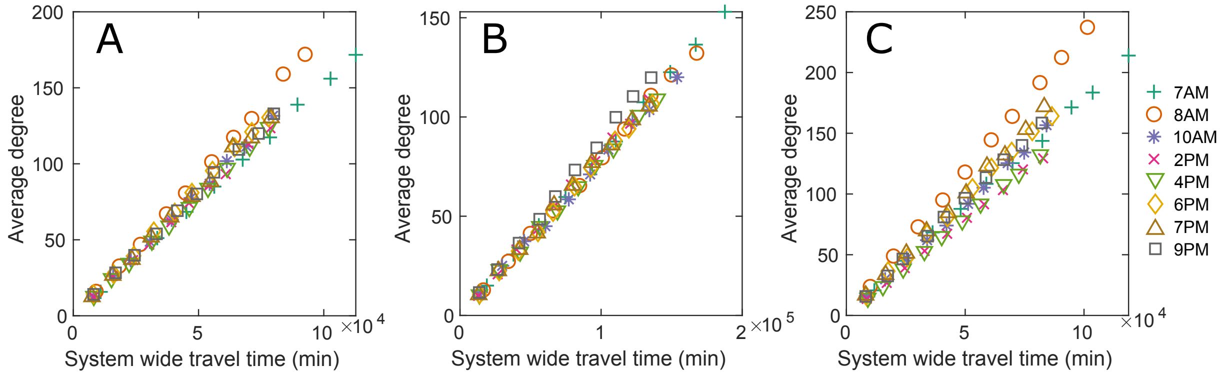

Figure S8: Relationship between the number of contacts per individual and the total system travel time. (A) Guangzhou, (B) Shanghai and (C) Shenzhen. The change of travel time reflects the increase in number of nodes from 500 to 5000 with an increment of 500 at each step.

where f_i and f_j represent the normalized trip flow traveling on OD pair i and OD pair j (number of nodes on OD pair i divided by total number of nodes in MCN). $\sigma_{i,j} = \frac{d_{i,j}}{maxd_{i,j}}$ refers to the standardized contact duration between the two OD pairs. In this regard, each entry of Q measures the pairwise contagion strength and row i of Q therefore gives the level of correlation of OD pair i with all other OD pairs. And the correlation depends on both the demand level as well as the contact duration.

Given the correlation matrix Q, we next extract the top n eigenvalues of Q as $\lambda_1, \lambda_2, ..., \lambda_n$, and we define similarity index as the standard deviation among the top n eigenvalues:

$$s = \sqrt{\frac{\sum(\lambda_i - \bar{\lambda})^2}{n}} \tag{27}$$

where $\bar{\lambda} = \sum \lambda_i / n$ refers to the mean of the eigenvalues. With the normalization of trip flow and standardization of contact duration, we restrict s to lie between 0 and 1.

The idea of similarity index is related to the principal component analysis of the correlation matrix, where the trace of the correlation matrix measures the total variance and the top n principal components seek to maximize the variance. The standard deviation among the top n eigenvalues therefore measures the differences in the total contribution to the total variance of each component, and hence reflects the trip differences among passengers. In particular, if those trips are totally uncorrelated and the trips, then the eigenvalues are all of the same value and the standard deviation among them is simply 0. An example in metro system is that the demand are evenly distributed among all OD pairs and these OD pairs have no overlapping segments to enable contacts. On the other hand, if some of the trip pairs are highly correlated, we should have few eigenvalues of value much higher than others, which gives rise to the large standard deviation. An extreme case in the metro system is that all travelers leave from the same origin to the same destination so that these trips are perfectly correlated and the standard deviation is therefore 1. The correlation matrix for metro systems is of size $N^2 \times N^2$ (N here is the number of stations) and we do not need to compute all N^2 eigenvalues. Instead, based on empirical observations, we find that eigenvalues drop quickly to nearly zero and we therefore set $n = 200$.

S6 First and second moment of node degree in MCN

Here we develop the first and second moment of the MCNs based on the generation model. We also derive an approximation of the largest node degree in a given MCN. These help to gain further insights on the degree distribution of the MCNs with an increasing number of nodes.

S6.1 First moment $< k >$

From the generation model for MCN we have:

$$p(k) = \sum_{i=1}^{N} e^{-Mw_i} \frac{(Mw_i)^k}{k!} p(w_i) \tag{28}$$

And we can calculate the average degree of the MCN as:

$$< k >= \sum_{k=1}^{K} p(k)k = \sum_{k=1}^{K} k \sum_{i=1}^{N} e^{-Mw_i} \frac{(Mw_i)^k}{k!} p(w_i) = \sum_{i=1}^{N} p(w_i) \{ \sum_{k=1}^{K} k e^{-Mw_i} \frac{(Mw_i)^k}{k!} \} \tag{29}$$

When $K \to \infty$, the summation of discrete degree can be replaced with the integration:

$$\sum_{k=1}^{K} k e^{-Mw_i} \frac{(Mw_i)^k}{k!} = \int_0^K k e^{-Mw_i} \frac{(Mw_i)^k}{k!} dk = Mw_i \tag{30}$$

where $M_w i$ is the mean of the binomial distribution of M trials and w_i rate of success. And we therefore have:

$$< k >= \sum_{i=1}^{N} p(w_i) M w_i \tag{31}$$

Note that $p(w_i) = p(t_i)$ represents the probability density function for human mobility in metro network, which we find to be approximated by an exponentially decaying tail. We consider that

$$Mw_i = \alpha(N-1) t_i^{\gamma_t}, \quad p(t_i) = b e^{-t_i/\lambda} \tag{32}$$

Then

$$< k >= \sum_{i=1}^{N} \alpha(N-1) t_i^{\gamma_t} b e^{-t_i/\lambda} \approx \int_0^{t_{max}} \alpha(N-1) t^{\gamma_t} b e^{-t/\lambda} dt \tag{33}$$

Where the integration gives

$$\int t^{\gamma_t} b e^{-t/\lambda} dt = -b \lambda^{\gamma_t+1} \Gamma(\gamma_t + 1, \frac{t}{\lambda}) + C \tag{34}$$

where $\Gamma(m, n)$ is the upper incomplete Gamma function with $\Gamma(m, n) \to 0$ if $n \to \infty$, and $\Gamma(m, 0) = \Gamma(m)$. This suggests that

$$< k >= \alpha(N-1) b \lambda^{\gamma_t+1} \Gamma(\gamma_t + 1) \tag{35}$$

which suggests that the **average degree of MCN is linearly proportional to the number of nodes in the network**.

S6.2 Second moment $< k^2 >$

In addition, we can also calculate $< k^2 >$ as

$$< k^2 >= \sum_{k=1}^{K} p(k) k^2 = \sum_{i=1}^{N} p(w_i) \{ Mw_i (1 - w_i + Mw_i) \} \tag{36}$$

where $Mw_i(1 - w_i + Mw_i)$ represents the second moment of the binomial distribution. Following the same procedure for deriving $< k >$, we arrive at the expression of $< k^2 >$ as

$$< k^2 >= \alpha^2 (N-1)^2 b \lambda^{2\gamma_t+1} \Gamma(2\gamma_t + 1) + O(N) \tag{37}$$

This implies that the variance of MCN scales quadratically to the increase in number of nodes. These results explain the divergence of $< k^2 >$ with $N \to \infty$.

S6.3 Max degree node k_{max}

To estimate the maximum degree in MCN, let we consider the probability that

$$\begin{aligned} \int_{k_{max}}^{\infty} p(k) dk = 1 - p(k_{max}) &= 1 - \sum_{i=1}^{N} p(w_i) \sum_{k=1}^{k_{max}} e^{-Mw_i} \frac{(Mw_i)^{k_{max}}}{k_{max}!} \\ &\approx \sum_{i=1}^{N} p(w_i) e^{-Mw_i} \frac{(Mw_i)^{k_{max}+1}}{(k_{max}+1)!} \\ &\approx \frac{b B^{k_{max}+1} \Gamma(k_{max}+2)}{(k_{max}+1)!(B + 1/\lambda)^{k_{max}+2}} \end{aligned} \tag{38}$$

where $B = \alpha(N-1)$.

And for the maximum degree, we expect that

$$\int_{k_{max}}^{\infty} p(k)dk = \frac{1}{N} \tag{39}$$

so that there is one node that is within the range $[k_{max}, \infty]$. This condition suggests that

$$\frac{B^{k_{max}+1}}{(B+1/\lambda)^{k_{max}+2}} = \frac{1}{Nb} \tag{40}$$

By taking the natural log on both sides, the equation simplifies to

$$\begin{aligned} k_{max} + 1 &= \frac{ln(Nb) - ln(B+1/\lambda)}{ln(B+1/\lambda) - ln(B)} \\ &\propto \frac{1}{ln(1+1/(\alpha\lambda(N-1)))} \\ &\propto \alpha\lambda(N-1) \end{aligned} \tag{41}$$

where for the last step we make use of the Taylor series expansion for log values

$$ln(1+1/(\alpha\lambda(N-1))) = \frac{1}{\alpha\lambda(N-1)} + O(1) \tag{42}$$

This result indicates that the **maximum degree of the MCN is linearly proportional to the number of nodes in the network**.

References

[1] Gaode Map API. `http://map.amap.com/subway/`, 2018. Accessed: 2018-09-30.

[2] Guangzhou metro timetable. `http://cs.gzmtr.com/ckfw/fwsj/`, 2018. Accessed: 2018-09-30.

[3] Shanghai metro timetable. `http://service.shmetro.com/en/hcskb/240.htm`, 2018. Accessed: 2018-09-30.

[4] Shenzhen metro timetable. `http://www.szmc.net/page/eng/time_table.html`, 2018. Accessed: 2018-09-30.

[5] Duncan J Watts and Steven H Strogatz. Collective dynamics of 'small-world'networks. *nature*, 393(6684):440, 1998.

[6] Sami Hanhijärvi, Gemma C Garriga, and Kai Puolamäki. Randomization techniques for graphs. In *Proceedings of the 2009 SIAM International Conference on Data Mining*, pages 780–791. SIAM, 2009.

[7] Deepayan Chakrabarti, Yang Wang, Chenxi Wang, Jurij Leskovec, and Christos Faloutsos. Epidemic thresholds in real networks. *ACM Transactions on Information and System Security (TISSEC)*, 10(4):1, 2008.

[8] Gene H Golub and Charles F Van Loan. *Matrix computations*, volume 3. JHU Press, 2012.

[9] Mark EJ Newman. Clustering and preferential attachment in growing networks. *Physical review E*, 64(2):025102, 2001.

[10] Frank J Massey Jr. The Kolmogorov-Smirnov test for goodness of fit. *Journal of the American statistical Association*, 46(253):68–78, 1951.
